# Acknowledgment to Reviewers of *JPM* in 2021

**DOI:** 10.3390/jpm12020168

**Published:** 2022-01-27

**Authors:** JPM Editorial Office

**Affiliations:** MDPI AG, St. Alban-Anlage 66, 4052 Basel, Switzerland

Rigorous peer-reviews are the basis of high-quality academic publishing. Thanks to the great efforts of our reviewers, *JPM* was able to maintain its standards for the high quality of its published papers. Thanks to the contribution of our reviewers, in 2021, the median time to first decision was 21 days and the median time to publication was 39 days. The editors would like to extend their gratitude and recognition to the following reviewers for their precious time and dedication, regardless of whether the papers they reviewed were finally published:
A. N. M. Mamun-Or-RashidLarry FliegelAbdeldjalil OuahabiLars HeggelundAbdo E. KabarritiLászló Csaba MangelAbdo Karim TourkmaniLatha PalaniappanAbdul QayyumLaura Ambra NicoliniAbdulhameed Al-GhabkariLaura Ancuţa PopAbdullah Al MarufLaura B. ScheinfeldtAbdulraheem AlshareefLaura CercenelliAbdurrahman I. IslimLaura GianottiAbhishek KulkarniLaura Katharina SieversAbhishek Kumar SinghLaura MarabiniAbhishek ThavamaniLaura SerraAbhishek VartakLaurence BianchiniAbraham Acevedo-ArozenaLaurent MetzingerAchim TemmeLaurent SuppanAdam GuastellaLaurentiu PirteaAdam KernLaurie M. ElitAdam M. DubisLawrence BrodyAdam MiddletonLawrence Shih Hsin WuAdam WichniakLea BenturAdamantia LiapikouLea TimmermannAdelaida AvinoLeandro Balzano-NogueiraAdile OrhanLeandro Rodrigues SantiagoAdina BrahaLech B. DobrzańskiAdina RoceanuLeena P. BharathAdrian CovicLeigh E. SzucsAdrian CurtoLeila JamalAdrian T. PressLeila Motlagh ScholleAdrian V. LeeLena HirtlerAdriana RusuLeonardo ManciniAdrien FlahaultLeonardo OggianoAfona ChernetLeonardo PellicciariAfroditi BoutouLeonardo TanziAfroditi NanouLeonie Marloes VogtAfsane RiaziLeopoldo FornerAfua Adjeiwaa MensahLetizia GiampietroAgata Schramm-LucLia VerhoefAgnes JanovszkyLiana PlesAgnieszka BanasLiang-In LinAgnieszka Kolasa-WołosiukLicínio MancoAgnieszka LatosinskaLi-Jen LeeAgnieszka Lecka-AmbroziakLikwang ChenAgnieszka MlynarskaLiliana PatruccoAgnieszka PiaseckaLiliana RogozeaAgnieszka SabiszLiliana TutaAgnieszka SiejkaLiping ZhangAhmad MirzaLiron RozenkrantzAhmad Raza KhanLirong WangAhmad SayasnehLisa BossAida MetoLisa SalvatoreAinhoa Ulibarri OchoaLi-Ting KaoAiping LiuLluís ColomoAkanksha SinghLoka PenkeAkashi FujitaLoredana AmorosoAkinori MitaniLoredana MarcuAkinori NakamuraLorena Ortega MorenoAkira MonjiLorenz TheilmannAkriti SrivastavaLorenza RimassaAkshara RaghavendraLorenzo RidolaAkshata R. NaikLorenzo RusticoAlain ThierryLorenzo VinanteAlan BittlesLori BanksAlan L. MyersLori Baugh LittlejohnsAlan WuLori RiceAlba García-CidLu QiAlba Timón-GómezLubomir SkladanyAlbert ComelliLuca AmbrosioAlberto BarbieriLuca BedettiAlberto BeharLuca FalzoneAlberto FerrareseLuca FreddiAlberto García-ZamalloaLuca GiannellaAldo Cordova-PalomeraLuca GilibertoAldona KasprzakLuca MarinelliAlejandro Sanz-ParisLuca RevelliAleksandar VišnjićLuca Salvatore MengaAleksandra KołtuniukLuca SebastianelliAleksandra KristoLuca TestarelliAleksandra Rajewska-RagerLucaciu RoxanaAleksandra WilkLucas H. P. BerntsAlessandra BorsiniLucia ScarabelAlessandra MarescaLuciana CacciottolaAlessandra PullieroLuciana CaenazzoAlessandro AlaimoLucilla ParnettiAlessandro BarboneLucrezia LaterzaAlessandro BlasimmeLuigi Alberto PiniAlessandro CeliLuigi Angelo VairaAlessandro CrosioLuigi BarreaAlessandro Gonzalez SalernoLuigi BennardoAlessandro GozzettiLuigi GianturcoAlessandro GranitoLuigi LoscoAlessandro MazzoniLuigi Maria LaroccaAlessandro NotaLuigi MeccarielloAlessandro RussoLuigi MinafraAlessandro SquizzatoLuigi R. CeciAlessandro TonacciLuigi RigacciAlessandro VittoriLuigi SantacroceAlessia ArcaroLuigi TritapepeAlessio BellatoLuigi VetrugnoAlessio GasperettiLuis Antonio Marinas-PardoAlessio GerussiLuis Mariano EstebanAlessio MylonasLuis SousaAlessio PaladiniLuísa Bandeira LopesAlexander ArltLuisa JordaoAlexander BerezinŁukasz ŁapajAlexander GlushakovŁukasz M. MilanowskiAlexander N. SavostyanovLukasz SzczerbinskiAlexander PodgorsakŁukasz SzczukowskiAlexander W. EckertŁukasz ZapałaAlexandra BlakemoreLu-Ting KuoAlexandra DamerauLyudmila Bel’skayaAlexandra NikakiM. Bruce MacIverAlexandra PerricosM. CellinaAlexandra Ripszky TotanM. Leontien Van Der BentAlexandre AnesiMabel BladesAlexandre Gonzalez-RodriguezMac GardnerAlexandre JoostenMaciej Iek SkrzypczakAlexandrina MunteanMaciej TarnowskiAlexandros RovasMădălina Maria DiacAlexandru MesterMaddalena MartellaAlexey MelnikovMagali HumbertAlexey MeshkovMagdalena BaymakovaAlexios Fotios A. MentisMagdalena Chottova DvorakovaAlexios PanoutsopoulosMagdalena Krajewska-WłodarczykAlfonso Javier Ibáñez-VeraMagdalena Paplińska-GorycaAli AkalinMagdalena StaszczakAli BakhshinejadMagdalena SzymanskaAli FarahaniMaged N. Kamel BoulosAli H. Husseen Al-NuaimiMahadevan SeetharamanAli ZarrabiMahboubeh YazdanifarAlice GiontellaMahmut Cerkez ErgorenAlice VassiliouMaiko MatsushitaAlicja UrbaniakMaite Teresa BetésAlmudena Buesa-EstellézMaïté VerreaultAlphus D. WilsonMaja Ortner HadžiabdićAlvaro Gallego-MartinezMalcolm HorneAlyssa LaForme FissMałgorzata GałeckaAmadeus DobrescuMałgorzata JamkaAmalia CorneaMałgorzata KujawskaAmanda TolandMalik BisserierAmandine RoviniMamdouh El-ShishtawyAmaniel KefleyesusMan Fai LawAmar ParvateMana RaoAmbica ParmarManabu ShigeokaAmelia BarcelliniManal FawzyAmelia CataldiManiselvan KuppusamyAmelia FilippelliManish ShahAmelia Maria GamanManisha TripathiAmer HarkyMansoureh TatariAmerian D. SonesManuel Albornoz-CabelloAmir El HassaniManuel GentiluomoAmir SherifManuel Hermida PrietoAmir VarediManuel PestanaAmit Kumar SahaManuel RequenaAmjad KhanManuel Sánchez-SanchezAmlan ChakrabortyManuel ScimecaAmritlal MandalMarcella TazzariAmy L. StarkMarcello GuarnieriAmy MessersmithMarcello MocciaAna AlfirevicMarcin A. JedrykaAna BudimirMarcin KowalskiAna Margraida AraújoMarco AielloAna Miklavčič VišnjevecMarco AlvesAna Nieto-RuizMarco CarboneAna Rita FariasMarco CarliAnamaria BechirMarco CostanziAnand GururajanMarco DelsanteAnandharajan RathinasabapathyMarco ManfrediniAnanth ShankarMarco MascarellaAnastasia MpakaliMarco MascittiAnastasia StoopsMarco MiloneAnastasios E. GermenisMarco OliveraAnberitha Anberitha MatthewsMarcos Arturo Martínez-BanaclochaAnca Lucia PopMarcos Díaz-GayAnca MesarosMarcus KrügerAnders Rosendal KorshoejMarek PetrášAndjelka KovačevićMargaret DeAngelisAndrás BikovMargarete SteinerAndrás RabMargarida F. B. SilvaAndre NybergMargarida VieiraAndrea AbateMargarita A. SazonovaAndrea AlexandreMargherita CerroneAndrea FabboMargherita EufemiAndrea GeorgiouMargherita MaranesiAndrea JorgensenMargherita ProsperiAndrea MilziMargie ClapperAndrea MurrayMargo TurnbullAndrea PacificiMaria Angeles Cuadrado-CenzualAndrea PiccioniMaria Bueno MarinasAndrea QuattroneMaría Camacho EncinaAndrea RoccaMaría Carmen Sánchez-GonzálezAndrea SambriMaria ContaldoAndrea SpallarossaMaría Cristina Martínez-FernándezAndrea TrombettaMaría Del Carmen Ortuño-CostelaAndrea VergallloMaria EspositoAndreas EbneterMaria Fátima PaulinoAndreas KalogeropoulosMaria FaviaAndreas SchmutzlerMaria FranziniAndreas WreeMaria Graciela CastroAndreia M. SilvaMaría José Gutiérrez CoboAndreja FigurekMaria Margherita RandoAndreu CatalaMaria MeloAndrew G. MarshallMaria MempinAndrew HarkinMaría Mercedes Suarez-CunqueiroAndrew L. HongMaria Michelle PapamichaelAndrey PlotnikovMaria MilettaAndrey S. GlotovMaria MoschoviAndrey ZamyatninMaria Pino-YanesAndrzej ObojskiMaria Rosaria De MiglioAndrzej PawlikMaria Rosaria RicciardiAneta Cymbaluk-PloskaMaria SukkarAneta PrzepiorskiMaria Teresa VietriAngel L. PeyMaría Yolanda Castellote-CaballeroAngel M. Garcia-LoraMarialucia GalloriniAngela PecoraroMariamena ArbitrioAngelika IllgMarian TraynorAngelina MeirelesMariana Bastos-OreiroAngelo NaselliMariana Cardoso DiogoAngelo RuggieroMariana FloriaAngelos HalarisMariane Maffei AzumaAngioletta LasagnaMarianna DelussiAnguraj SadanandamMariano S. PedanoAnh NguyenMarie Øbro FosbølAnil Kumar JonnalagaddaMarie T. KrügerAnimesh AcharjeeMarieke J. H. BegemannAnirudh SharmaMariela PadillaAnita Kuldeep MehtaMarija GamulinAnita QuintanaMarija MihajlovicAnja Kathrin JaehneMarijn SpeeckaertAnja WesselyMarike LeijsAnjana BairagiMarina Gálvez-PeraltaAnna BaranMarina SagudAnna EnglerMarina V. DunaevaAnna GiovanettiMarinos G. SotiropoulosAnna MajerMario BauerAnna MnatsakanovaMario PacilliAnna PapiezMarisel Romina TuttobeneAnna SauerbierMarius ZahanAnna SkalniakMariusz KusztalAnna StaniszewskaMariusz SikoraAnna StarzyńskaMarjeta TerčeljAnna WaśniewskaMark A. GreenAnna Wołoszyn-DurkiewiczMark A. HirschAnnalisa CarlucciMark BrownAnnalisa LevanteMark D. TricklebankAnnalisa PatriziMark L. DreherAnnalisa PernaMark R. De HoraAnne ChiaoMark S. TaylorAnne W. BeavenMarko KumrićAnne-Cécile PaepegaeyMarko MarhlAnnelien DuitsMarko VuletićAnne-Sophie WoznyMarkus BlaessAnni-Maija LindenMarkus DuechlerAnoop KumarMarkus KrebsAnouk EmadaliMarkus SchoenbergAnthony CrimarcoMarkus StraussAnthony HerbertMarkus WilhelmiAntoine DrieuMaroof AlamAntoinette C. Van Der KuylMarta BertaminoAnton KorbutMarta BistronAntonella RivaMarta ChecchiAntonietta BernardoMarta Garcia-ClementeAntonietta GiganteMarta GrodzikAntonija TadinMarta MakowskaAntonio CentofantiMarta PadovanAntonio De LeoMarta StarnoniAntonio GalloMarta ToczekAntonio Giovanni SolimandoMartijn ScherrenbergAntonio González-BulnesMartin BauerAntonio MacciòMartin BergerAntonio Palazón-BruMartin Cornelius JordanAntónio QueirósMartin DyrbaAntonio RagusaMartin Schmidt-HieberAntonio SalarMartina KroppAntonio SalsanoMartina MaggiAntonio SanzoMartina SilvestriAntonio SerranoMartina SmolićAntonio Simone LaganàMartyna Kasper-JędrzejewskaAntonios A. KoutalosMarwan A. S. BukhariAntti KämppiMary Anna VenneriAnupam AichMary J. KastenAnurag SharmaMary L. WhiteAparna ShindeMaryam Mobed-MiremadiArend Derk Jan Ten HarkelMaryam NakhjavaniAri Vander WaldeMasa SasagawaArindam BhattacharjeeMasahiro HayashiArjun AthreyaMasahiro SugimotoArkadiusz SzarekMasakazu TanakaArmando AliuMasaki OkamotoArmando CaseiroMasao OtaArmin FinkenstedtMasaoki KohzakiArne KienzleMasaru TanakaArtor Niccoli AsabellaMasayuki FujiiArturo CesaroMasayuki NakamuraArturo OrlacchioMasayuki UrabeArun K. IyerMassimiliano MirabellaArun SamiduraiMassimo D’AgostinoAshini WeerasingheMassimo GeunaAshleigh HockingMassimo ImazioAshley E. RossMassimo LibraAshok PatowaryMassimo PapaleAshvin R. JaiswalMassimo TusconiAthanasios AnagnostisMatea Nikolac PerkovicAthanasios AnastasiouMatea ZajcAtsumi NittaMatej Sapina SapinaAtsuo KikuchiMateusz WiniarczykAtsushi SakamotoMathias TessonAttayeb MohsenMathilde Body-MalapelAttilio AM BianchiMatija RijavecAudrey AdjiMats SjöströmAugusto Garcia-AgundezMatteo Della PortaAureliusz KolonkoMatteo Luigi Giuseppe LeoniAvital SterninMatteo TagliapietraAxel KünstnerMatteo ZampiniAyaho YoshinoMatthew B. LanktreeAyataka FujimotoMatthew CookeAyelén ToroMatthew D. StrubAyoub Al-JawaldehMatthew KoehlerAyumi SugiuraMatthew Meng Yang LeeAzadeh A. T. BorojeniMatthias BuechterBaiba JansoneMatthias D. HoferBalamurugan RamatchandirinMatthias GüntherBaoming LiuMatthias KochBaoyin ZhaoMatthias MillesiBarbara AdamikMatthias SchwabBarbara AltieriMattia D’AgostinoBarbara Bowles BieseckerMattias PaulssonBarbara JasiewiczMaurizio CasarrubeaBarbara KabonMauro GiuffrèBarbara Mangweth-MatzekMauro RagoneseBarbara Sosnowska-PasiarskaMauro TognonBarbora KonečnáMax Lennart EcksteinBart PijlsMaximilian DietrichBartosz LeszczyńskiMaximilian RudertBartosz PułaMaximilian SeidlBashkim KadriuMayra PaolilloBeata Kasztelan-SzczerbinskaMd Abdul HakimBeata Sarecka-HujarMd Jamal UddinBeatriz MerinoMd Masud ParvezBeatriz SastreMd. Mohaimenul IslamBebiana CondeMd Monirul IslamBeenu Moza JalaliMeg SimioneBehnam RashidiehMegan L. UhelskiBehnoush Abedi ArdekaniMegan RobertsBelinda SchwerinMeghan Underhill-BlazeyBenilde CosmiMegumi UchidaBenjamin EssayaghMehdi NassiriBenjamin Q. DuongMehmet M. AltintasBenjamin VoellgerMehmet SarierBenoit MiottoMehraboon IraniBenson Chellakkan SelvanesanMehran DadrasBenussi LuisaMehwish AnwerBeom Kyung KimMeir NitzanBernadette MurphyMeir Tibi MarmorBernadette O. FernandezMelinda KolcsárBernard NaafsMelissa Agsalda-GarciaBernard PossidenteMelvin R.Pete HaydenBernd SchweizerMeng WuBernhard H. F. WeberMengxi ShenBernhard UehlekeMercedes Bueno-CampañaBernward LauerMerry Lynn Noelle McDonaldBert-Ram SahMette RaunkiaerBhadran BoseMevlüt ÇelikBhanwar Lal PuniyaMezentsev AlexandreBhavin VasavadaMichael A. JacksonBhavuk GargMichael B. FischerBianca GoemansMichael BohlmannBianca HanganuMichael BouvetBianca Karla ItariuMichael C. WiestBianca NitzscheMichael D. FreemanBignardi MarioMichael DeelBimal ChaudhariMichael EskinBin ChenMichael H. GoldBin WangMichael HutterBjoern TitzMichaël LaurentBjörn FalkenburgerMichael R. KozlowskiBlanca Ribot SerraMichael UhlinBo HuMichael VoulgarelisBo YangMichael W. KrainerBodnar RichardMichaela A. BotiBogdan Marian SorohanMichaela ButrynBogdan SoceaMichail DiakosavvasBoris B. NiraulaMichał BieńkowskiBożena BukowskaMichał BorysBozena Romanowska-DixonMichał BurdukiewiczBrach PostonMichał GinsztBradley EdelmanMichal Inbar-FeigenbergBradley FergusonMichal LinialBradley QuonMichal NowickiBradley SchaeferMichal SarulBrent SieskyMichał StasiowskiBrian Andrew Van TineMichala ShortBrian F. O’DonnellMichalina J. GóraBrian K. FoutchMichel Kabamba NzajiBrian PieningMichel KranendonkBrisa FernandesMichela FerrucciBrisa S. FernandesMichela RugoloBritta BerglundMichele D’AttilioBruce AronowMichele Di CosolaBruna PanizzuttiMichele FinottiBruno GeierMichele LanzaBruno MégarbanéMichelle Whirl CarrilloBuckley McCallMichiel E. R. BongersBulat RamazanovMiguel A. OrtegaByeong-Ho JeongMiguel A. SanzByoungjin ParkMiguel BritoByungchul OhMiguel BurgosByung-Kwan SeoMiguel Castelo-BrancoC. Jane WelshMiguel Mayo-YáñezCaius ConstantinescuMiguel OrtegaCalvin EiberMihaela BadeaCamille CarrollMihaela MoscaluCamlyn MasudaMihaela ZegreanCan ÖztürkMihai MusteataCandan HizelMihajlo JakovljevicCaren J. FrostMiika MartikainenCarey MatherMika LehtoCarla MinoiaMikael CohenCarla PalmaMikel SanchezCarlo CamerlingoMikhail MilchenkoCarlo M. PetriniMiki KushimaCarlo PalomboMikolaj DabrowskiCarlo PapponeMila A. EmeraldCarlos RochaMilena Adina ManCarmen BurteaMin Young SeoCarmen CasanovasMinchan GilCarmen RinaldiMing-Jen LeeCarmine IzzoMing-Shao TsaiCaroline GurvichMingyi ZhangCaroline HeckmanMinliang LiuCarpén TimoMinna StoltCatarina GodinhoMiquéias Lopes-PachecoCatarina Pereira-LeiteMiriam MarquésCaterina BensiMiriana D’AlessandroCaterina BrighiMirko ManchiaCaterina LeddaMiro JukićCathriona KearnsMiroslav BartakCelia Ruiz-SánchezMiroslav IlićCengiz TürkseverMiroslav VaverkaCesare DanesinoMiroslava NedyalkovaChaeuk ChungMirosław J. SzczepańskiChaido SirinianMiroslaw JanowskiChandrakanth EdamakantiMirza PojskicChandrashekhar HonraoMirza PojskićChang-Hoon ChoiMitali TambeChang-Myung OhMitja MitrovicChang-Shen LinMittal JasoliyaChanpreet SinghMizuho NishioChanyang MinMohamed Abdel-NasserChao ChenMohamed Adel HammadChao MaMohamed Al-FatimiChaoxing LiuMohamed AliCharles MarksMohamed KhettabCharles SabbaghMohamed MeghilCharu KothariMohammad AlamChen LiaoMohammad Ehsanul KarimCheng-Chieh YenMohammad Muzaffar MirChengguo WeiMohammed IlyasChenghui LiMohammed S. SalahudeenCheng-Shun LuMohd Parvez KhanChenyu LiMohd Saleem DarCherie B. NauMohit KumarCheuk Lun LiuMohsen KhosraviChia-Che LeeMona MathewChia-Feng LuMonica CalasansChia-Hung ChouMónica FernandesChia-Hwa LeeMonica Livia GheorghiuChia-Ju LinMonica RienzoChian Ju JongMonika CsókaChiara BelliaMonika FurkoChiara Di RestaMonika Gudowska-SawczukChiara GabbiMonika LejmanChiara Maria MazzantiMonika Pazgan-SimonChiara MussiMonika SzeligaChiara SorberaMonique J. RoobolChiara VillaMoon-Soo LeeChien-Feng LiMoritz HamannChien-Hung LeeMoritz MirnaChien-Hung LiaoMosiewicz JerzyChih JunMotiur RahmanChi-Nien ChenMubin TarannumChin-Sheng HungMuhammad RiazChris PlanqueMuhammad Saad KhanChris WallMuhammad Sohail KhanChristel DanielMukesh Kumar SriwastvaChristelle Darrieutort-LaffiteMukesh VermaChristian BarbatoMulugu V. BrahmajothiChristian BrendelMun-Gwan HongChristian ChiamuleraMurali Krishnan KolikondaChristian KonradsMurugabaskar BalanChristian L. LaboisseMutsumi IijimaChristian WirajaMychel MoraisChristian ZanzaMyles FaithChristian ZurthMyrto PapamentzelopoulouChristina BehrN. T. MoldogazievaChristine Espinola-KleinNabil EidChristine PatchNachiket DharkerChristoph HartmannNadeem QureshiChristoph ReinhardtNagendra N. Dudi-VenkataChristophe BadieNajmaldin SakiChristophe BattailNalini ChintalapudiChristophe CombetNamrata KhuranaChristophe MassetNamshin KimChristophe OrssaudNanae HarashimaChristopher HillNancy Monroy-JaramilloChristopher J. YoungNanda MandavaChristopher KobierzyckiNansu ZongChristos ChatzikyrkouNao FujimoriChristos DamaskosNaoki MatsumotoChristos GeorgiouNaoko KandaChrong-Reen WangNarayan ShivapurkarChunbin ZouNarendra K. SinghChung-Ta LeeNaresh Doni JayaveluChun-Jen HuangNatale MussoChunxu GaoNatalia BaulinaChunzhang YangNatalia FilippovaCinzia AllegrucciNatalie DeuitchCinzia CasuNatalja EigelieneCiprian RoiNatalya A. ShnayderClaire BilloteyNatasha FranklinClaire LeconteNatasha J. PetryClara GreenNathalie Baeza-KalleeClaudia BrognaNathan K. EvansonClaudia MehedintuNathanael J. YatesClaudia PisanuNathaniel HendrixClaudia SaghedduNauck MatthiasClaudio FerranteNaushad Ali JungleeClaudio FiocchiNeamtu BogdanClaudio LuchiniNelly Schulz-WeidnerClaudio MarchettiNelson YeeClaudiu MorgovanNiall ColganClemens HöbausNiamh M. TroyClement ChungNibedita PatelClément LahayeNicholas CoupeClement Richard BolandNicholas V. RescinitiColleen A. CampbellNicki NiemannConal CunninghamNicola Alessandro IacovelliConcetta SantonocitoNicola MagnavitaConsolato M. SergiNicola MartiniCordelia Estevez-CasellasNicolae GicaCorey NislowNicolae MironCorina BocsanNicolai Van OersCorina Marilena CristacheNicolaus FriedrichsCormac OwensNicoleta AntonCorrado De VitoNicoleta Stefania MotocCorrado TagliatiNidaa MikaïlCosimo CumboNiels BergslandCraig HodgesNiels WestergaardCrescenzio SciosciaNihar J. MehtaCristian DeanaNikhil Suresh BhandarkarCristian FurauNikola StefanovićCristian MirvaldNikolaos A. StavropoulosCristina Anna Maria Lo IaconoNikolaos MachairasCristina AntinozziNikolaos PapanasCristina L. RonchiNikolaos TsamprasCristina NadaluttiNikolaos V. MichalopoulosCristina SmolenschiNikos KaracapilidisCrystal FengNina F. Tabachnik SchorCsilla PesznyákNina Milutin GašperovCsongor KissNina ModerauCynthia AristeiNina SperberCynthia Marie-ClaireNiranjan BaluCyrille GrandjeanNiroz Abu-Saleh ZoabiCyrus MotamedNirupa SachithanandanDacia Dalla LiberaNishant PuriDagmara KłopotowskaNishel Mohan ShahDaiki TSUJINitesh MittalDaisuke NishizawaNitika KumariDa-Liang OuNoam NissanDalius JatuzisNora HenriksonDamiano FantiniNorbert F. KissDamu TangNorio YamamotoDan Cristian RaduNoriyoshi SawabataDan RossignolNorman R. WilliamsDanial RoshandelNoura AlsufyaniDaniel BäckerNowicki AndrzejDaniel C. MaloneNuno A. SilvaDaniel CronaNuno Rodrigues Dos SantosDaniel García IglesiasNuno ValeDaniel Gilbert WeberNuria Gago-LópezDaniel HagenfeldOana Falup-PecurariuDaniel HeiseObul Reddy BandapalliDaniel HernandezOctavian NeagoeDaniel MeltersOfir KorenDaniel MorgansteinOgechi IkediobiDaniel OuyangOgnjen BarcotDaniel P. PotaczekOke GerkeDaniel P. ZalewskiOlaf MichelDaniel PredaOlga BureninaDaniel RamsköldOlga DolgovaDaniela BartoșOlga GordeevaDaniela Elena SerbanOlga KorczeniewskaDaniela GasparottoOlga SisoevaDaniela Valeria MinieroOlga SukochevaDaniele CastellaniOliver H. KrämerDaniele FanaleOliver HaaseDaniele La ForgiaOliver W. HakenbergDaniele UrsoOmar JanehDaniella HowOndrej KrejcarDanijela Ristić-MedićOnofrio LaselvaDanilo MenichelliOrsolya VargaDaqing MaOscar CampuzanoDarcy PoolerOscar LaoDarin ZertiOu-yang ChaoDario RahelićOvidiu MituDario SiniscalcoOvidiu SirbuDario TrapaniOzan DikilitasDaryl PritchardPablo Ramos-GarciaDavid A. RizzieriPablo S. Luna-LozanoDavid Alan RizzieriPablo ZubiaurDavid BeversdorfPa-Chun WangDavid CokerPalle Duun RohdeDavid DrozekPalma SolanoDavid EdvardssonPanagiotis KassanosDavid FergusonPanagiotis PeitsidisDavid GibsonPanayotis ThanosDavid Gonzalez De CastroPao-Huan ChenDavid GrahamPaola DefilippiDavid HormuthPaola IratoDavid Hung-Tsang YenPaola QuaresimaDavid Moreno MolinaPaolo FerrariDavid PagliaPaolo ImmovilliDavid PoppersPaolo PelliccioniDavid Ramiro-CortijoPaolo RadiceDavid RobergePaolo TrerotoliDavid T. YeungParaskevi KatsaounouDavid WuParichoy Pal ChoudhuryDavid ZweikerParisa K. KargaranDavide BizzocaPark Heung-WooDavide CavagnettoParthi SrinivasanDavide DonatiParul VarmaDawei LiPascal DjiadeuDawid PołapPascale GauthierDawid SzwedowskiPasquale ParibelloDeborah CholonPasupathi SundaramoorthyDeborah NovelliPatrícia A. MadureiraDebra De Assis RosaPatricia Miranda AzpiazuDeena WalkerPatrick DunnDeepak Kumar KhajuriaPattraporn SatitsuksanoaDeepanshu JainPaul B. FitzgeraldDelia Mirela TitPaul CarrierDelphine TuotPaul E. EngelhardtDenisa MarginaPaul LacazeDennis AdjepongPaul TucanDennis W. T. NilsenPaulina DumnickaDerrick Matthew BuchananPaulina TrybekDer-Yuan ChenPaulo LopezDespoina ManousakiPaulo MartinsDhamodharan VenugopalPaulo PaixãoDhananjay YadavPavan MadanDhelal Al-RudainyPavel LobachevskyDiana M. VerboomPavel MadonovDiana MartinsPavol ZelinaDiana Tordesillas-GutierrezPawel GajdzisDiane MègePaweł GutDidier DuclouxPaweł KrukowDiego Francesco CalvisiPaweł KrzyżekDiego Tlapa-MendozaPaweł PietkiewiczDijana ZadravecPedro BerjanoDimiter AvtanskiPedro Henrique Lopes SilvaDimitrios DimitroulisPedro ZapaterDimitrios KouroupisPeeyush RanjanDimitrios PatouliasPei-Lun (Perry) KuoDimitrios T. PapadimitriouPei-Yi ChuDimitris E. GiakoustidisPei-Yuan SuDimitris TatsisPeka ChristovaDinakaran ThirumalaiPengcheng WangDinesh K. ThekkinkattilPengfei DongDinh Toi ChuPetar OzretićDionysios TafiadisPetca Razvan-CosminDisha SharmaPeter C. M. van de KerkhofDivya BhagirathPeter D. SenterDivya KalraPeter DrainDmitri NepogodievPeter HindmarshDmitriy NiyazovPeter J. EggenhuizenDmitry B. ZorovPeter JarcuskaDmitry TworowskiPeter N. SchlegelDoga KuruogluPeter PikoDomenico Di RaimondoPeter PolveriniDomenico LioPeter SandwallDominika ZającPeter TörökDomizia VecchioPetra Anna GolovicsDon GreenPetros MourouzisDonald Max SmithPetros SountoulidesDonald R. Van DevanterPhilip R. MuskinDong SongPhilipp MayerDong-Guk PaengPier Luigi AntignaniDong-Seok GwakPierfrancesco FrancoDong-Wook KangPierluigi RinaldiDorela Shuboni-MulliganPiero RicchiutoDorin IonescuPierpaolo Di MiccoDorina StoicanescuPierre-Yves RisoldDorota Bielinska-WazPietro CacialliDorothea HarrisonPietro CaliandroDorothee SchneiderPietro ScicchitanoDorothy KesslerPim A. de JongDragana KomnenovPing MuDragos MedianPing-Chieh PaoDragos SerbanPing-Ho ChenDrew NassalPing-Hsun WuDrozdstoj St. StoyanovPiotr DobrowolskiDubravka Švob ŠtracPiotr GabryelDusica StamenkovicPiotr GaleckiDustin J. HinesPiotr SobaniecE. David G. McIntoshPiotr WaleckiEddy LangPlinio CirilloEdoardo MonfriniPo-Chang HsuEdoardo StaderiniPo-Yu FongEduard Pedemonte-SarriasPo-Yu KaoEduardo CelentanoPrabhu MathiyalaganEduardo Fuentes QuinterosPradeep V. KadambiEduardo López-UrrutiaPrakash RaiEduardo PuellesPranabananda DuttaEdvard GalićPranjal SarmaEdward ModestinoPranshu SahgalEdward N. HarrisPrasanth PuthanveetilEdward WylegalaPratikkumar DesaiEdyta ŁuszczkiPraveen KumarEfstratios-Stylianos PyrgelisPrimrose FreestoneEftychia DemeroutiPriscila KrollEhsan HabibiPritam SadhukhanEhtesham ArifPritish MondalEinar ArnbjörnssonPritmohinder S. GillEitaro KodaniPriyamvada JayaprakashEkaterina TolstayaPriyanka KhareEl Hadji M. DioumPriyanka SabharwalEleanor E. R. HarrisPriyanka VyasElena BencúrováPriyankar DeyElena BertelliPrzemysław KołodziejElena Escolano-PérezPutty-Reddy SudhirElena KaschinaPuy GarrastachuElena LevantiniQi ChenElena OlivettiQi FangElena PoddubskayaQIAN DUElena PrietoQian-Rong QiElena RapizziQimin HaiElena SizikovaQin GaoElena TarcaQin Xiang NgElena-Adelina TomaQingbo LiuEleni MavrogonatouQingyu ZhouEleonora Bielawska-BatorowiczR. M. HermannElham SajjadiRachel DryerElias CasulaRachel NadifElin ChorellRachel Wah Shan ChanElisa Eugenia CórdobaRadhia M’kacherElisa María Garrido-ArdilaRadu AlbulescuElisabeta BotezRafael López-LópezElisabetta BertelliniRafael Moreno-Gómez-ToledanoElisabetta PennacchioliRafael Ríos-TamayoElissavet KardamiRaffaele PalmirottaElizabeth B. TorresRaffaele SerraElizabeth KukielkaRaffaella CascellaElizabeth S. DylkeRaffaella CerrettiElizabeth SokolRaghavendran ParthaElizabeth VafiadakiRaghu SinhaEllen LeichRahul Dev JayantEllen McGinnisRahul Prasanna KumarElric ZweckRaja GanesanElzbieta KlimiecRajendra D. BadgaiyanEmanuela CollaRajendran JC BoseEmidio ScarpelliniRajesh Tota-MaharajEmilia Modolo PintoRajeshwary GhoshEmilio González-JiménezRajinder DawraEmma EspinosaRalf BraunEmma ShtivelmanRalf DresselEmrin HorgusluogluRalph A. BundschuhEndre CzeiterRalph GeorgeEnrica Teresa TandaRam SamujhEnrico CapobiancoRam SharmaEnrico CassanoRama Sashank MadhurapantulaEnrico GranieriRamesh NarayananEnrico MiniRamesh PeriasamyEnrique ColadoRami M. AlzhraniEric De GrootRami S. KantarEric R. BravermanRami ZewailEric S. HoRan JingErick S. LebrunRanjeev Chrysanth PulleErik KudelaRares CraciunErik SmedlerRasa SabaliauskaitėErika MendozaRatko PrstačićEsayas Kebede GudinaRatnadeep MukherjeeEsperanza Begoña García NavarroRaúl Capote-PuenteEsteban Obrero-GaitánRavindra KolheEsther TantsisRavindra KumarEstíbaliz JiménezRecep AvciEugen DivjakReece Andrew SophocleousEugenia BezirtzoglouReginald M. GorczynskiEugenia ScaricamazzaReinhard Schwartz-AlbiezEugenio AgugliaRemo CampicheEva BagyinszkyRenata De Almeida CoudryEva LietoRenata Novak KujundžićEvens Idil ApakRenato CuocoloEwa FiedorowiczRene ChunEwa GrzybowskaRene HurlemannEwa JablonskaRené Klinkby StøvingEwa StachowskaRené R. H. HartensuerEwa WolińskaRengasayee VeeraraghavanEwelina DziurkowskaRenuka RamanEwelina MaculewiczRiad HaddadEwoud SchuitRicardo Castro AlvesEyal SagivRichard Bayley PaiseyFabian TroschelRichard E. FryeFabian WeykampRichard J. StarkFabio PastorinoRichard L. AutenFabio SallustioRichard P. BoardmanFabiola PaiarRichard RokytaFabrizio GentileRick StevensFahad QuhalRie Eriksen BechsgaardFandi IbrahimRifat AhmedFang TongRiitta SuhonenFangfang QiaoRika MaruyamaFarah R. ZahirRikhav P. GalaFaris MujezinovićRishi Man ChughFarnoosh Abbas-AghababazadehRita KissFausto AssandriRita PavasiniFederica BelloneRita PolitoFederico GulluniRitin SharmaFei MoRiuko OhashiFelice CrocettoRivka KessnerFelipe AlconchelRobbert W. SchoutenFelipe VilellaRobert BestFelix HaglundRobert EckenstalerFelix Javier Jiménez-JiménezRobert FriemuthFélix Javier Jiménez-JiménezRobert M. VerdijkFeng HeRobert PassierFerdinando AgrestaRoberta GraneseFerdinando FranzoniRoberta PalmourFerenc SiposRoberto ChimenzFernanda RodriguesRoberto CirocchiFernando De CastroRoberto Díaz PeñaFernando HernanzRoberto GirelliFilipe PortelaRoberto GramignoliFilippo MaselliRoberto GrassiFilippos TriposkiadisRoberto Lo GiudiceFiona CousinsRoberto ManfrediniFiona LyngRocio Alejandra Chavez-SantoscoyFlora CimminoRocio Eiros BachillerFlorence BoissierRodica BălașaFlorence T. BourgeoisRodolfo CapannaFloriana Bulic-JakusRodrigo A. LoiolaForest FabienRoger E. ThomasFranca DipaolaRohitash JamwalFranca NataleRoman FleckFranca R. GueriniRomana VulturarFrances GardenRon BardinFrancesc Xavier AvilesRon H. N. Van SchaikFrancesca ArianiRona CheifetzFrancesca BruzzeseRonald B. BrownFrancesca BugiottiRonald James A. WandersFrancesca DiomedeRosa Anna VaccaFrancesca GelfoRosa Di LiddoFrancesca MagnoniRosa VallettaFrancesca ManniRosaria V. AbbrittiFrancesca SciontiRossana IzzettiFrancesca TrojsiRossana RoncatoFrancesca ZaraRostyslav BubnovFrancesco BennardoRoxana Maria NemeșFrancesco ChiarelliRoxana OanceaFrancesco GallettiRoxana Ramona OnofreiFrancesco MannelliRoy ZiegelsteinFrancesco MaseduRuben TorregrosaFrancesco PetrellaRui VitorinoFrancesco StilloRundk HwaizFrancisco Alcantud MarínRupesh K. MishraFrancisco J. AlcainRupesh ShresthaFrancisco J. MedranoRuslan DorfmanFrancisco Javier López RománRute SantosFrancisco Sáez-OrellanaRuth ChadwickFrancisco SampaioRuth JackFranco CervellatiRyan J. FarrFranco M. BuonaguroRyan MaysFrank Vinholt SchiødtRymantė GleiznienėFrank WolframRyon M. BatemanFranz HoelzlRyong Nam KimFranziska HoffmannRyuichi OhtaFranziska WelzelS WagnerFrauke StankeSabata MartinoFrédéric CoutantSabata PiernoFrederic TriponezSabin J. BozsoFrediano InzaniSabina Antonela AntoniuFurqan AzizSabina TangaroGabor MikalaSabine WrengerGabriela-Dumitrita StanciuSabrina PriclGabriele GhettiSabrina Rahman ArchieGabsik YangSachiyo MoritaGaetano GalloSaeed Ur RehmanGaetano IsolaSaeid KargozarGaetano PaoneSaeideh NozohouriGaetano TerranovaSafak Guel-KleinGaia BistulfiSafikur RahmanGajanan GhodakeSahil BajajGalina V. PortnovaSahil SharmaGang PengSaid ShamiehGannon C. K. MakSally Anne MortlockGanta Vijayx ChaitanyaSalma BuddasethGao ZhuangqiangSalomone Di SaverioGarima DwivediSalvador PérezGaryfallia PeperaSalvatore BocchieriGaurav GhagSalvatore CarusoGaurav V. SarodeSalvatore Giovanni VitaleGediminas GaidulisSalvatore GrisantiGeertruida Hendrika De BockSalvatore PastaGema MiñanaSalvatore ScaccoGeorge P. KostyukSamar IssaGeorge PatrinosSameh GehaGeorge PavlidisSami Abd ElwahabGeorge ValasoulisSamir GorasiyaGeorgios KotzalidisSamuel Peña-LlopisGeorgios PapathanakosSamuel TomczykGerard AnmellaSamuli EldforsGerard BerrySamy M. RiadGerassimos E. VoutsinasSanchita RauthGerd U. AuffarthSandeep SinghGerrit M. GrosseSandra CurinierGesche JürgensSandrine LauranceGhais KharmandaSangjin KimGholamali RahnavardSanjairaj VijayavenkataramanGholamreza DaryaborSanjay RaoGholamreza HaqshenasSanjiv NeupaneGiacomo CapponSankaran ChandrasekharanGiacomo GaribottoSanne W. Ten BroekeGiada PietrabissaSantiago CepedaGiampaolo PernaSantiago MoraGiampiero LeanzaSantos VillafainaGian Carlo PecorariSantosh KumarGian Paolo CavigliaSara BlaineGianfranco SpallettaSara Huerta-GonzálezGianina LucaSara M. FalzaranoGianluca FranceschiniSara PerettiGianluca IngrossoSarah BärGianluca RigatelliSarah M. NelsonGianmarco AbbadessaSarah Page HammondGianna DiPalmaSarbjit SinghGilad HalpertSascha K. ManierGina Maria CaetanoSatadru LahiriGiner-Soriano MariaSatoshi WashinoGionata FragomeniSatoshi YamaguchiGiorgia CapocasaleSavino SpadaroGiorgio BerlotSayeda Yasmin-KarimGiorgio I. RussoScott M. SchuetzeGiorgio MalpeliSead CrnalicGiorgio PolettiSebastian BöttgerGiorgio RussoSebastian KlobuchGiorgio Walter CanonicaSegundo Nicolas SeclenGiovanna FloridiaSeil KimGiovanni Battista PeregoSeiya YokoyamaGiovanni CasellaSeka LazareGiovanni TarantinoSeon-Cheol ParkGiovanni TisciaSerafin MoralesGiulia BIVONASergey V. KovalchukGiulia CarusoSergio GarciaGiulia DondiSergio MalutaGiulia FerrarazzoSergio OcchipintiGiulia MidenaSeth M. TarrantGiulia PulianiSeung-bae YoonGiuseppe BroggiSeungil RoGiuseppe ComentaleShahab HaghayeghGiuseppe De MicheleShahina PardhanGiuseppe LosurdoShaista MalikGiuseppe MagliettaShamroop Kumar MallelaGiuseppe MarroneShang-Ming ZhouGiuseppe SantarpinoShang-Yu WangGiuseppina MartellaShanmugarajan KrishnanGleb SlobodinSharad PurohitGloria PascualSharanjot SainiGonzalo J. RevueltaSharda KumariGowtham K. AnnarapuSharmilee GnanapavanGracia Castro-LunaSharon AndersonGrant McArthurSheeba JacobGreg EbertShekoufeh AlmasiGreg GibsonSheng ZhuGregory A. DanielsSheng-Dean LuoGregory BowdenSheng-Der HsuGregory PoonSheng-Hsuan LinGrzegorz PorebskiSheng-Min HsuGrzegorz R. JuszczakSheng-Yow HoGrzegorz ZielińskiShevach FriedlerGuang Yih SheuShiang-Fu HuangGuankui WangShibiao WanGudrun DandekarShigeyuki TakamatsuGuido KrenningShih-Yi LinGuido Santos-RosalesShike WangGuillaume E. CourtoyShin Jun ParkGuillaume HerbreteauShinji YamashitaGuillermo GomezShintaro SukegawaGuiomar Perez De NanclaresShirley M. TsunodaGunnar SchottaShiv VermaGunnar SchuetzShivshankar ThanigaimaniGuobin XiaShreoshi Pal ChoudhuriGuozheng WangShu-Chun LinGustavo GlusmanShu-Fen WuGuy RostokerShuhei ItoGyoergy KovacsShujiro OkudaGyozo SzolnokyShujuan ChenH. Miles PrinceShu-Ling TzengHadi MessihaShu-Yan LiHaidara AlmansourShyam MoreHaishu LinShyouichi HazamaHaitham JahramiSibte HadiHammam AlshazlySiddharth SunilkumarHamza El HadiSigve NakkenHan-Chao ChangSilke KrolHankyu LeeSilverii Giovanni AntonioHans Christoph LudwigSilverio RotondiHänsli ChristofSilvia De Miguel-BilbaoHans-Peter SimmenSilvia FilippiHao LiSilvia LiuHaosu ZhangSilvia M. Sanz-GonzálezHarinarayanan JanakiramanSilvia Sanz-GonzálezHaris MemisevicSilvio IontaHarissios VliagoftisSimão PinhoHazem WafaSimon ClarkHeather E. DanyshSimon CouillardHeather Gordish-DressmanSimon G. GregoryHeather M. HowerSimon J. FurneyHee-Geun JoSimon KasifHeiko BrennenstuhlSimon KirsteHeinrich GerdingSimona BorstnarHélder SpínolaSimona CavaluHelen TwohigSimona MitevaHelena IdborgSimona TeccoHelena JoséSimone KleissHelena L. A. VieiraSimone PernaHelmut KlockerSimran MaggoHemant GiriSin-Ho JungHengyao NiuSireesha ManneHenning ReisSiro LuvisettoHenrik GalboSitansu Sekhar NandaHenrique SilvaSiu Wai ChoiHenry LancashireSiwei ZhangHermann B. FrieboesSlava BergerHerwig GerlachSofia BhatiaHeyrim ChoSofia PereiraHideo WadaSofia S. PereiraHideya YamamotoSoichiro MurataHideyuki FukuiSokol TrunguHilde Kanli GaltungSokou RozertaHimanshu BatraSolomon AdamsHimanshu DeshwalSomaieh KazemnejadHimashu Narayan SinghSomasundaram PrasadhHinne A. RakhorstSomchai AmornyotinHipolito NzwaloSoňa NevšímalováHiroaki InoueSonam KumariHiroki TeragawaSongnan WenHirono IriuchishimaSonia Kaniuka-JakubowskaHiroshi Inui InuiSonja KlebeHirotaka IwakiSophia LionakiHiroyoshi Y. TanakaSøren MikkelsenHiroyuki IsayamaSoterios KyrtopoulosHiroyuki MoriSotirios ApostolakisHisao ImaiSouichi SuenobuHisham FansaSoumeth AbasseHitoyuki DaidaSoumyabrata MunshiHo TsoiSoumyajit D. RoyHon Lon TamSo-Youn KimHongcui CaoSoyoun UmHongliang HeSpyridon KanellakisHongwu WangSrba MijailovichHooshang LahootiSreenivasan JayakumarHorea FeierStanescu Ana Maria AlexandraHossein TaghizadehStanislav PantelyushinHotcherl JeongStefan Cristian VesaHsiang-Hsuan Michael YuStefan GrundHsiao Tien LiuStefan HohausHsin-Yao WangStefan KabischHsueh-Ju LuStefania NobiliHsu-heng YenStefania ScuriHua-Sheng ChiuStefanie Hessel-PrasHui WangStefano Francesco CrinoHuijun ChihStefano PinardiHulens MiekeStefano RauseiHumbert González-DíazStefano TuroloHun Soo ChangSten DreborgHwi-Dong JungStephan GerberHyoung-Yeon SeoStephan GrimaldiHyun KwonStephan SchererHyun-Ah KimStéphane DerruauHyung Joon JooStephane R. GrossHyung-Soo HanStephanie BaudHyun-jin MinStephanie DeLucaIan CampbellStephanie DuguezIan MorisonStephanie N. L. SchmidtIbtihal Al AmriStephanie SchipmannIevgen KoliesnikStephen LiggettIftikhar J. KulloStephen MullinIgnacio González-GarcíaStephen OpatIgor ElmanStergios BoussiosIlaria CampioniSteve Woosuk HurIlaria OttonelliSteven FeldmanIlaria UmbroSteven G. KovenIlir AgalliuSteven M. FrischIlona SadauskieneSteven M. HeatonIman M. TalaatSteven Paul NisticòIn Gyu SongStrippoli SabinoIndra SarkarStuart A. ScottInga Griskova-BulanovaStuart GrieveIngeborg BrouwerStuart RundleIngela E. TuressonSuba RajendrenIngrid Fricke-GalindoSucharu GhoshIngrid TonniSudheer RavuriInmaculada Conejos-SanchezSudhir Putty ReddyInna S. MidzyanovskayaSudipta BiswasIoana Roxana BordeaSufang LiuIoanina ParlatescuSu-Hyung HongIoanna DimopoulouSu-Jun LeeIoannis BellosSukh MakhnoonIoannis IliasSun ImIoannis TsamesidisSun Kwang KimIonela Ruxandra SfeatcuSuneel KumarIqbal MahmudSunetra SaseIrena NalepaSung Hea KimIrene AprileSung Soo AhnIrene M. VavasourSungmin KimIrfan KhanSungok ChangIrina SufaruSunil KarhadkarIrinel PopescuSun-Young KongIris SilvaSusan BaileyIrma Mahmutovic PerssonSusan RichterIryna DykunSusan SnyderIsaac RheeSusan SumnerIsabel MercaderSusana B. BravoIulia AndrasSusannah J. KingIvan BrakSusanne RospleszczIvan BruknerSushama SivakumarIvan CorazzaSven FlemmingIvan NombelaSyed Haris OmarIvan RadojaSygkliti-Henrietta PelidouIván Santolalla-ArnedoSylviane MullerIvana SamarzijaSzczepan PaszkielIvana SutejSzczepan PiszczatowskiIvo BoškoskiSzymon JanczarIvo DobrevSzymon SiecińskiIwona Jarocka-KarpowiczTadayoshi BesshoIwona SidorkiewiczTaehoon KoIzabela ChmielewskaTae-Joon KimIzabela Korona-GłowniakTaha Koray ŞahınIzabela ZakrockaTahereh JavaheriIzumi KajiTaiji NagaokaJacek BilTaishi HataJacek CapalaTakaaki InoueJacek R. WilczyńskiTakaaki SenbonmatsuJacek StępniewskiTakafumi KatoJae-Hong KimTakahiko MorotomiJae-Seon SoTakahiro NemotoJai N. PatelTakahiro SekiJaime Font De MoraTakahiro SodaJakov MilićTakane Kikuchi-UedaJakub KaźmierskiTakanori YasuJakub RokTakashi MatsusakiJakub S. GąsiorTakashi OnoJakub SlupskiTakeji SakaeJames BluettTakeo NakadaJames D. St LouisTakeshi SasakiJames Kevin HicksTakshashila TripathiJames MainprizeTaku AokiJan BilskiTalida Georgiana CutJan BuikemaTamara Nikuseva MarticJan DamoiseauxTamas HaideggerJan KrogTamás Kovács-ÖllerJan MejzlikTang Her JaingJan Niklas Petry-SchmelzerTania López HernándezJan Philipp RadtkeTanja Grubić KezeleJan PithaTanja KunejJan PlockTanja StocksJane M. CarringtonTanzila SabaJangik Ike LeeTao ChenJanhavi NagwekarTara Clinton-MchargJanko DiminićTarec Christoffer El-GalalyJános SándorTaro TakeuchiJanoš TerzićTatsuo KandaJanusz BlasiakTeet SeeneJanusz KarasińskiTeodora Alexa-StratulatJarosław J. SakTeresa CardosoJasenka WagnerTeresa Sanchez-GutierrezJasminka Ilich-ErnstTessandra StewartJason A. HortonTetsufumi TakahashiJavier Conde ArandaTetsuya HirataJavier EspinoThanapong ChaichanaJavier Ochoa-ReparazThao P. Ho-LeJavier Sanchez GalanTheis Skovsgaard ItenovJayson V. PagaduanThelma SpindolaJeanine RoodhartThemistoklis TsatalasJean-Marc SellalTheodora KatsilaJean-Michel LecerfTheodore KolivakisJee Soo ParkTheodoros AslanidisJeffrey M. SundstromThierry AlcindorJeffrey M. WitkinThirunavukkarasu SathishJeffrey R. StrawnThomas DittmarJeffrey TowbinThomas DuflotJeffry B. LansmanThomas JouveJennifer B. McCormickThomas KerkhofsJennifer D’SouzaThomas MawhinneyJennifer GlausThomas MohrJennifer MytychThomas MüllerJennifer SugaThomas NührenbergJenny QueThomas P. McCoyJens P. DreierThomas Patrick LillicrapJens Von Der GrünThomas TapmeierJeong-Hoon LeeThor BechsgaardJeonghyun AhnThorsten RudroffJere PaavolaThwe HtayJérémie GautheronTiago DiasJeremy W. ProkopTianhua NiuJerome BeckerTianjun WangJerzy JuśkiewiczTiffany A. MooreJesse J. KellerTiffany M. DelhommeJessica L. ReynoldsTill Jasper MeyerJesús PastorTilman CalliessJia Ping WuTilmann BochtlerJiafen HuTim HohmannJialin MengTim HulsenJianqiu XiaoTim R. GlowkaJianzhen XuTimmy LiJidong SungTimo MeineJiffin K. PauloseTimo MühlhausJijia WangTimo SchinkoetheJijun HuangTimothy J. RobinsonJin WeiTimur SellmannJin ZhangTina Tičinović KurirJindrich KopecekTingting ShiJing HaoTinhinane FaliJing PangTirthankar SinhaJing ZhangTissa WijeratneJin-Ming WuTiziana CiarambinoJin-Rong ZhouTobias DombrowskiJitendra KanaujiyaTobias RomeykeJitka VeldemaTodd KnepperJoana DamásioTolga ÖzmenJoana MesquitaTomasz HachajJoanna CytarskaTomasz HirnleJoanna DepciuchTomasz UrbanowiczJoanna KonopińskaTomasz WypychJoanna KowalskaTomasz ZielińskiJoanna Petryka-MazurkiewiczTomaszewski MichałJoanna Saluk-BijakTommaso ErcoliJoanne DickinsonTommaso LombardiJoão Carlos RibeiroTomoyuki AbeJoão Gama MarquesToni PollinJoao LoboToralf ReimerJoão MesquitaToru YagoJoão Miguel Marques Dos SantosToshiaki OnitsukaJoão Valente DuarteToshiaki SaidaJochen G. SchneiderToshihiko TashimaJochen SchneiderTsai-Tsen LiaoJochen WalterTsuyoshi SugiuraJody DushayTuan XuJoe JamesTudor Lucian PopJohannes FesslerTuong-Vi NguyenJohannes MosbacherTuulikki Sokka-IslerJohn DowneyTzong-Yuan WuJohn J. HalperinUdayabhanu JammalamadakaJohn J. LimaUdo JeschkeJohn J. SidtisUgo FalagarioJohn Patrik BurkhardUlf D. KahlertJohn W. LaceUlf GunnarssonJohn W. NicholsonUlf SchminkeJohn WatertonUlrich JuliusJohn YovichUlrich KellerJolanta E. LosterUlrich LinsenmaierJolanta JaworekUlrich RonellenfitschJonathan BenzaquenUlrika Snygg-MartinJonathan C. SchislerUmar ShafiqueJonathan E. SearsUmberto CaterinoJonathan RoydsUmesha BoregowdaJongmin SimUrs A. MeyerJörg KleeffUrszula CytlakJörg MarotzUrvish PatelJorge AzevedoUte GschwandtnerJorge Luis EspinozaUtpal Kumar MondalJørgen A. RungbyUwe BlunckJosé A. CañasUwe SiebertJosé AgundezValentina BessiJose Antonio Camacho-CondeValentina BulloJose Antonio Hurtado-SánchezValentina DiniJose Antonio Lopez-EscamezValentina LanteriJosé Augusto SimõesValentina VengelieneJose BernalValentyn OksenychJosé BragancaValeria BertagnoloJosé Carmelo Adsuar SalaValeria VezzoliJose Felix. Moruno ManchonValerian DormoyJose Ignacio LopezValerio MaisJose L. Gómez-UrquizaVanessa ZagoJose Luis Pérez-CastrillónVangelis G. ManolopoulosJosé Luis Romero-BejarVarut VardhanabhutiJosé Luis SanzVasiliy DevyatkinJosé Luis Vázquez-NogueraVassiliy TsytsarevJosé Manuel CifriánVenkata Lokesh BattulaJose Maria MarimonVenugopal GundaJosé Miguel Seguí-RipollVera FaustinoJose Palacios CalvoVěra FrankováJosé Paulo AndradeVered RazJosé Raduan JaberVeria VacchianoJose Sanchez-AlcazarVeronika AleksandrovychJosé-María Sánchez-GonzálezVicente Romo PérezJoseph NassifVictor E. OrtegaJoshua ChewVictor GarciaJosip A. BorovacVictor Marcos-GarcesJosip TomićVictor ValderrabanoJosipa BukicVictoria L. Halperin KuhnsJoy MitraVictoria MacBeanJu Han KimVijay AvinJuan Francisco Rodríguez-TestalVikram K. MulliganJuan Francisco Sánchez-SolerViktor HamreforsJuan Lorenzo TerrasaViktor ReichertJuan MunizVilma PetrikaiteJuan P. RodrigoVincent CrennJuan Pablo RigalliVincent PonsJuan Rodríguez MansillaVincenza ConteducaJuan Sebastián Sandoval ArévaloVincenzo Manuel MarzulloJUANA SERRANO LOPEZVincenzo OcchipintiJuerg KesselringVioletta Opoka-WiniarskaJules DesmeulesVirendra ChaudhriJulia BujanVishnuprabu Durairaj PandianJulia Guerrero-GironésVita GolubovskayaJulian PohlanVito Andrea CapozziJulie SwartzendruberVito TerlizziJulien AncelVitoantonio BevilacquaJulio Plaza DíazVivek ManivelJulio Ricarte FilhoViviana NocitiJulio Ruiz-PalmeroVjekoslav PeitlJumpei SaitoVlad PadureanuJun FangVladimir M. MilenkovicJune Young ChoiVladimír ŠubrJunehawk LeeVladimir TrkuljaJung-Wook KimVladimir VladimirovJunko OkuyamaVolker J. SchmidtJurgen DittmerVucenik IvanaJu-Seop KangWalaa NasryJustin M. WattsWalid D. FakhouriJustin TseWalter J. FlapperJustyna GodosWalter KolchJustyna MiszczykWaqar RashidK. Ray ChaudhuriWaseem LoneKa Ki David WongWasim A. DarKa KimWei Chiao ChangKah Kheng GohWei LiKai WangWei NiKai-Wen HsuWei-Hsun YangKaja Blagotinšek CokanWeikuan GuKalra Rajkumar SinghWeiliang QiuKamal Kant SahuWen LiKamil GillWen LinKarel KotaškaWen-Chin HuangKarem A. CourtWen-Hui FangKaren Hirsch-ErnstWenping GongKarine BreckpotWenqiang ZhangKarl RösslerWenxiu ZhaoKarl-Arne JohannessenWenying LuKarlena Lara-OteroWenyue SunKarol Rawicz-PruszyńskiWeslania Viviane NascimentoKarthik UppuluryWesley R. BarnhartKashif RasoolWilliam DuddyKasper StokbroWilliam N. HaKatada SayakoWilly HauzerKatarina Trebušak-PodkrajšekWinnie S. Y. TanKatarzyna HolcmanWioletta MędrzyckaKatarzyna IżykowskaWito RichterKatarzyna KakarekoWitold WoźniakKatarzyna Komosinska-VassevWojciech BorawskiKatarzyna Kwiecień-JaguśWojciech JelskiKatarzyna LeszczynskaWolfram WeckwerthKatarzyna Mocny-PachońskaWoo Jong KimKatarzyna Skórzyńska-DziduszkoWoo-Baek ChungKatarzyna WalkiewiczWuwei RenKaterina GrafanakiXiajun ZhouKaterina K. NakaXiaodong WangKatharina AlackXiaohu HuangKatharina MartiniXiaomei CongKatharine BrighamXiaowen ZhangKatharine M. LodgeXiaoyan WangKatherine AlpaughXiaoyin XuKatherine J. E. SattlerXikun HanKathleen A. R. SchildrothXin CuiKathleen BradyXin JinKathleen DenisXingjuan ChenKathrin BlagecXu Dong ZhangKathryn A. PhillipsXuan LiKathryn D. BakerXudong HuangKathy MartynXuehua XuKatia PaneXuming MaoKatlyn E. BrownYae KanaiKatrina E. FurthYafei WangKaty De Paco MatallanaYagaung ZengKaustuv BhattacharyaYamin Tauseef JahangirKazimierz KuliczkowskiYanfei ZhangKazuaki TokodaiYang ChenKazuhiko HashimotoYang MeiKazutoshi MurataYannick SaalbergKazuya InokiYanwu XuKei TobiumeYaojiong WuKeiko MatsubaraYasuhiro MatsumuraKeissy SousaYasuhiro MikiKeith J. StineYasuhito TakahashiKeittisak SuwanYasuo ImaiKeizo AnzaiYasuyuki TokinagaKelly R. GreveYawei LiKelsey CookYee Ming LeeKelvin Ian AfrashtehfarYeongho KimKenji FujiwaraYet H. KhorKenji YokoiYeu SuKenneth BlumYftach GepnerKenneth HettieYi-Chiung HsuKenneth P. H. PritzkerYihong GuanKenneth R. FosterYi-Hung ChiangKenneth S. RamosYing HuKeno-Kyrill BressemYing LiKensuke KumeYingpeng LiuKentaro InamuraYisong TanKentaro MatsuiYitang SunKentaro YamashitaYi-Yen LeeKerry MillsYoav KohnKerry RenwickYohan NguyenKeun-Yeong JeongYong AiKe-Vin ChangYong Qin KohKevin DarbyYong-Zhang LuoKezia GaitskellYookyung BooKhaled E. AhmedYoo-Ri ChungKheng Siang NgYosep ChongKian Fan ChungYoshiaki ZaizenKilian WeigandYoshifumi SaijoKim E. NicolsYoshihiro NodaKim KampenYoshihiro NoguchiKimberly BusseyYoshiko MatsudaKimberly McCordYou-Lin TainKiran UpadhyayYounjae OhKisha PiggottYounsook Anna YeoKiTani Parker LemieuxYousif SubhiKiyoshi TakagiYoussef DaaliKlára DaďováYousuke NakaiKlaske Van KammenYuan LyuKlaus BønnelykkeYuan TangKlaus Gottlob MüllerYue ShengKlaus RadermacherYugo AshinoKok Hian TanYuichi IshikawaKomuraiah MyakalaYuichiro HatanoKonda Reddy KarnatiYuji SakaiKonrad S. JankowskiYuji ShimizuKonstantin SlavinYuki NakamuraKonstantinia AlmpaniYuki SaitoKonstantinos EvangelouYuki TanisakaKonstantinos KompotisYukinori MaruoKonstantinos SapalidisYuliang WuKonstantinos SidiropoulosYunfei LiKornelia Kuźnik-TrochaYun-Ju ChenKosuke YoshidaYURI BattagliaKotaro OgawaYusuke YoshiokaKrishna Chaitanya ThandraYuting TanKrishna Kishore UmapathiYuval EbensteinKristen M. WardYu-Wei WuKristen W. CarlsonYves LussierKristian SchneiderZack ShanKristina KuhbandnerZack Y. ShanKrupa NavalkarZaid TahaKrzysztof CzamaraZara SteinmeyerKrzysztof J. CzarnockiZarin ZainulKrzysztof KuciaZbigniew HeleniakKsenija Rener-SitarŽeljko VerzakKuan-Hao TsuiZenon PogorelićKun LiZeyun YuKunal DhimanZhaoning WangKunwei WuZheng Zachory WeiKyle B. WilliamsZhenya TangKyoung KimZhipeng LiuKyousuke TanakaZhipeng TaoKyung-Han LeeZhiqiang HuKyung-San MinZhiqun XieL. J. ValentijnZhiwei WenLaisa Liane Paineiras-DomingosZhonghua SunLaith AlzubaidiZijuan LiuLakshmi PrabhuZohaib KhurshidLamin B. ChamZoltan HeroldLampros PapadimitriouZoltán HeroldLanfranco PellesiZsolt GállLarisa AnghelZulqarnain BalochLarisa PinteZuzanna Nowicka


